# Chondromalacia in Lipedema: The Sarcopenic–Valgus Cascade That Keeps Getting Missed

**DOI:** 10.7759/cureus.95299

**Published:** 2025-10-24

**Authors:** Alexandre C Amato

**Affiliations:** 1 Vascular Surgery, Amato - Instituto de Medicina Avançada, Sao Paulo, BRA

**Keywords:** chondromalacia patellae, dynamic knee valgus, lipedema, pes planus (flatfoot), sarcopenia

## Abstract

Knee pain in women with lipedema is frequently misattributed and undertreated. We outline a biomechanical and inflammatory cascade linking systemic adipose dysfunction, anabolic resistance, and thigh-predominant sarcopenia to dynamic knee valgus, plantar arch collapse, altered gait, patellofemoral malalignment, and ultimately chondromalacia patellae. We integrate synovial-adipose crosstalk and the high prevalence of generalized joint hypermobility as amplifiers of joint loading. This framework supports a practical, staged approach that couples symptom control with progressive, targeted strengthening and gait retraining. Rather than treating the knee in isolation, addressing the cascade may reduce pain and improve function.

## Editorial

Knee pain in women with lipedema is too often misframed as an isolated patellofemoral disorder. In our Brazilian population study, 58.1% of women who screened positive for lipedema reported knee pain, which is evidence that this symptom is common and clinically meaningful rather than incidental. The mistake is reading the knee in isolation. Lipedema is a systemic, inflammatory disorder of loose connective tissue that reshapes load sharing across the lower limb and blunts muscular adaptation. In practice, patellofemoral morbidity typically unfolds along a coherent sequence: disease-related anabolic resistance limits strength gains; thigh-predominant sarcopenia emerges, especially in the quadriceps and hip abductors; dynamic genu valgum develops during weight-bearing tasks; the plantar arch progressively collapses with overpronation; the center of mass lowers and gait mechanics shift; hip and pelvic stabilizers are overworked; patellar tracking deteriorates; chondromalacia follows. Fat debulking alone cannot reset this chain; we must diagnose and treat the entire kinetic sequence rather than a single link. Given the 12.3% prevalence of probable lipedema among Brazilian women, the stakes for getting this right are substantial [1-6].

Lipedema inflammation impairs muscle adaptation

Lipedema tissue is not inert. Human studies show stage-dependent adipocyte hypertrophy, interstitial fibrosis, and chronic low-grade inflammation within subcutaneous fat-features that plausibly blunt hypertrophic signaling and contribute to anabolic resistance. Clinically, women with lipedema demonstrate lower lower-limb strength than BMI-matched women with obesity, suggesting disease-specific myofunctional compromise rather than deconditioning alone. This is the spark that lights the cascade [1, 2].

Thigh-predominant sarcopenia feeds dynamic valgus

**Figure 1:**
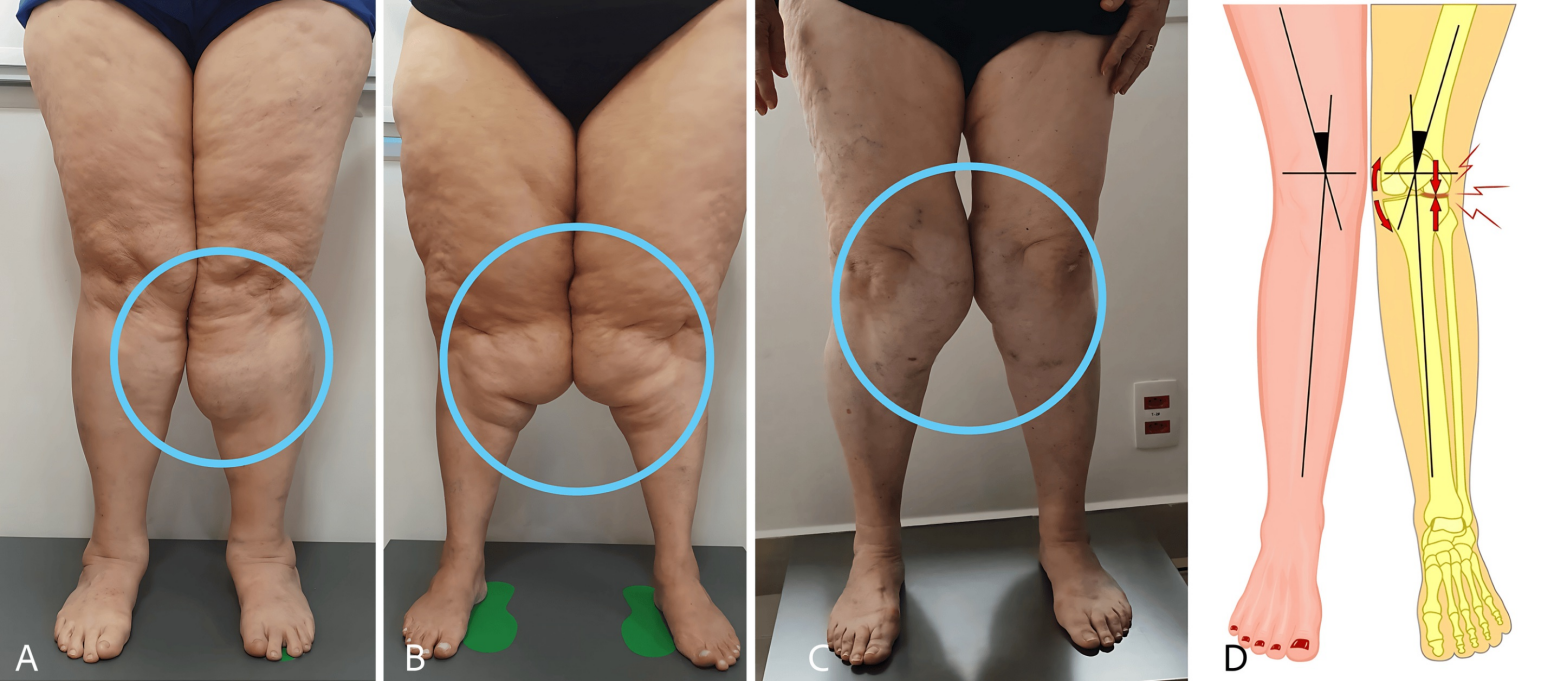
Peri-knee lipedema bulk and dynamic valgus mechanics (A–C) Frontal, quiet-standing photographs of three different adult women with lipedema, highlighting medial-thigh and peri-patellar adipose accumulation (blue circles). A prominent subpatellar fat pad can limit terminal knee flexion and bias frontal-plane mechanics toward dynamic knee valgus; subtle forefoot abduction/overpronation is also visible. All clinical photographs were obtained by the authors and are published with written informed consent for open-access publication. (D) Author-created schematic of genu valgum illustrating medial knee collapse and the resultant patellofemoral contact/stress vectors. © Aksana Kulchytskaya / Dreamstime.com, Image ID 121714824. Used under license.

Quadriceps and hip-abductor weakness are prime drivers of dynamic knee valgus (DKV), the medial collapse of the knee during weight-bearing tasks. Contemporary meta-analyses and kinematic studies link DKV to higher patellofemoral joint stresses and symptom exacerbation. In lipedema, medial-thigh bulk, pain, and soft-tissue impedance further bias the limb into adduction, reinforcing valgus moments and patellar maltracking during gait and stairs-precisely the loading pattern that accelerates chondromalacic change [7, 8]. This sarcopenic-valgus sequence, with its key modifiers and downstream consequences. See Figure 1 for clinical examples and a schematic of valgus mechanics.

Local joint-fat crosstalk (reactive synovitis → lipedema flare)

When patellofemoral maltracking progresses to chondromalacia, the joint capsule commonly mounts a reactive synovitis. This intra-articular inflammation increases local cytokine and neurogenic signaling, which, by contiguity through richly innervated, edematous subcutaneous tissue, can amplify inflammation in the overlying lipedema fat, driving pain hypersensitivity and favoring focal adipose deposition. In these cases, the patient’s “lipedema knee pain” is often secondary to the joint’s synovitis rather than to adipose load alone. Clinically, intra-articular hyaluronic acid (IA-HA) has shown promise in reducing synovitis and, in parallel, attenuating localized lipedema symptoms adjacent to the knee; evidence remains preliminary and hypothesis-generating, but the signal is encouraging and warrants controlled trials [9-14].

Hypermobility: the force multiplier

Generalized joint hypermobility (GJH) is strikingly common in women with lipedema (reported in around or above one-half of patients) and is clinically captured with Beighton-based criteria and, when appropriate, the 2017 hypermobile Ehlers-Danlos syndrome (EDS)/hypermobility spectrum framework [15]. In a limb already weakened by thigh-predominant sarcopenia, excess end-range mobility with poor neuromuscular control amplifies frontal- and transverse-plane errors: femoral adduction/internal rotation, tibial internal rotation, and rearfoot eversion. The result is deeper dynamic knee valgus and greater overpronation, both of which heighten patellofemoral loads and accelerate chondromalacic change. Beyond mechanics, GJH is linked to impaired balance and reduced muscle endurance-practical reasons to prioritize control-over-range strategies: progressive strengthening (hip abductors/external rotators, quadriceps), intrinsic foot training, dorsiflexion mobility done safely, short-term arch support, and cautious use of end-range stretching. In short, hypermobility does not cause the cascade-but it widens the funnel through which it flows [15-18].

The foot is “spared”, but the arch is not

The feet are typically spared of lipedema fat deposition. Yet clinical cohorts and expert consensus increasingly describe functional foot change (overpronation and flatfoot) as the limb adapts to proximal weakness and valgus. Pronation couples with tibial internal rotation and femoral adduction, amplifying patellofemoral stress. Systematic reviews now associate a more pronated/flat foot posture with patellofemoral pain, ignoring the arch means missing a modifiable amplifier of knee symptoms [19-21].

Lowered center of mass and altered gait propagate pain up and down the chain

As the arch collapses and valgus increases, the center-of-mass trajectory and ground-reaction vector shift. Patients adopt antalgic patterns with reduced push-off, short step length, and pelvis drop; hip abductors, rotators, and core musculature are over-recruited to “rescue” frontal-plane control. The result is a familiar composite: anterior knee pain, lateral hip pain, and often plantar heel pain from chronic tensile load at the enthesis. High-load, progressive strengthening of the plantar flexor complex outperforms stretch-only paradigms in plantar fasciitis, underscoring that graded loading (not resting) is the medicine for diseased fascia and weak intrinsics [22, 23].

Why fat removal alone is not enough

Lipedema reduction surgery (LRS) has documented benefits-reductions in pain, improvements in stance, gait, and even valgus angles-when performed by experienced teams. But surgery subtracts tissue; it does not add strength, proprioception, or motor control. Without targeted rehabilitation to restore hip abductors/external rotators, quadriceps capacity, ankle dorsiflexion, foot intrinsic strength, and gait mechanics, the same valgus-pronation pattern reasserts itself-now acting on a lighter, but still unstable, limb. The right message is “debulk barriers, then rebuild capacity” [24, 25].

A practical clinical algorithm: treat the cascade, not the knee

In practice, clinicians should treat the cascade rather than the knee in isolation. The evaluation should confirm adipose phenotype and pain by staging lipedema and mapping tenderness and pain distributions [2]; assess strength and control with single-leg squat and step-down to elicit DKV while measuring hip abductor/external rotator strength and quadriceps endurance [7]; and evaluate foot posture and mobility using the Foot Posture Index or arch-height measures, ankle dorsiflexion, and signs of plantar fascial irritability [21]. Capacity should then be rebuilt with graded, disease-aware loading that emphasizes hip-centric reconditioning to correct femoral adduction/internal rotation in stance [8]; quadriceps loading with pain-limited, progressive open- and closed-chain exercise to restore patellar tracking and shock absorption [26]; and a foot-ankle program comprising intrinsic foot training, calf raises with metatarsophalangeal dorsiflexion (windlass engagement), and short-term arch support while strength accrues [22]. Gait should be retrained with attention to cadence, step width, and pelvic control, and hypermobility should be managed with control-over-range strategies common in lipedema rehabilitation [19]. Structured exercise, combining strength and aerobic modalities, remains first-line therapy for function and pain in lipedema [27]. As adjuncts and in appropriate sequence, LRS is reasonable when anatomical bulk or medial-thigh impingement blocks movement, with prehabilitation and an early return to progressive loading to exploit improved lever arms and lower pain [25]; patellofemoral care (taping, bracing, and education) should be integrated within, never substituted for, kinetic-chain rehabilitation, acknowledging that multimodal, capacity-building programs outperform single-modality approaches across patellofemoral pain syndromes [28].

**Figure 2:**
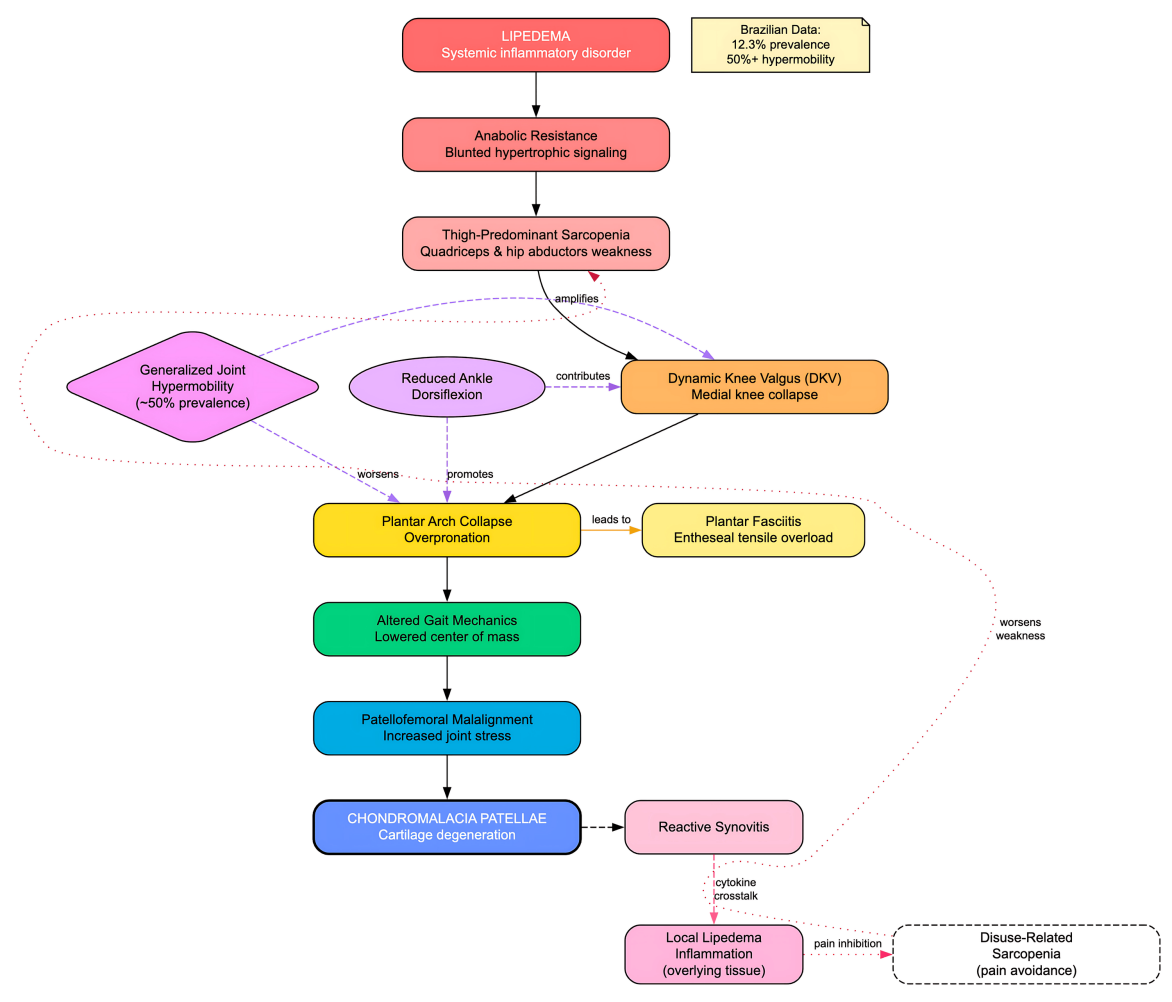
Lipedema–chondromalacia pathophysiology cascade Solid arrows depict the primary sequence from systemic inflammation in lipedema → anabolic resistance → thigh-predominant sarcopenia (quadriceps and hip abductors) → dynamic knee valgus (DKV) → plantar arch collapse with overpronation → altered gait with a lowered center of mass → patellofemoral malalignment → chondromalacia patellae [1-4, 7, 8, 10, 13, 14, 21, 28]. Dashed arrows indicate clinical modifiers—generalized joint hypermobility and limited ankle dorsiflexion [7, 8, 18, 21]. The side branch shows plantar fasciitis emerging from arch collapse [22]. The reactive-synovitis pathway illustrates joint–fat crosstalk, whereby synovitis may propagate inflammatory signaling to the overlying lipedema tissue. Dotted arrows denote a pain → disuse feedback loop that worsens sarcopenia [3, 4, 10, 13, 14]. In our Brazilian population study, 58.1% of women screening positive for lipedema reported knee pain, and the prevalence of probable lipedema was 12.3% [4]. DKV, dynamic knee valgus; PFJ, patellofemoral joint. Image credits: Author-created. Information synthesized from prior literature [1-4, 7, 8, 10, 13, 14, 18, 21, 22, 28].

Conclusion

Women with lipedema commonly present with knee pain that reflects an upstream cascade: anabolic resistance and thigh-predominant sarcopenia foster dynamic valgus and altered foot mechanics, which increase patellofemoral stress and provoke synovitis. Recognizing this pattern allows clinicians to pair local symptom control with progressive hip-quadriceps strengthening, dorsiflexion and foot-intrinsic training, gait re-education, and weight-bearing load management. When conservative care is optimized but function remains limited, lipedema-targeted interventions may further improve mobility and quality of life. Treating the cascade, not only the knee, offers the most durable path to improvement. Clinically, the mandate is clear: identify the cascade, calibrate loading, debulk when indicated, and rebuild every link.
